# Editorial: Arterial Aging and Age-Associated Arterial Diseases

**DOI:** 10.3389/fgene.2018.00444

**Published:** 2018-10-19

**Authors:** Mingyi Wang, Jiguang Wang, Cuntai Zhang, Gianfranco Pintus

**Affiliations:** ^1^Laboratory of Cardiovascular Sciences, National Institutes of Health, Biomedical Research Center, National Institution on Aging, Baltimore, MD, United States; ^2^Department of Hypertension, The Shanghai Institute of Hypertension, Ruijin Hospital, Shanghai Jiao Tong University School of Medicine, Shanghai, China; ^3^Department of Geriatrics, Tongji Hospital, Wuhan, China; ^4^Department of Biomedical Sciences, College of Health Sciences, Qatar University, Doha, Qatar; ^5^Biomedical Research Center, Qatar University, Doha, Qatar

**Keywords:** aging, arterial remodeling, proinflammation, hypertension, atherosclerosis

## Introduction

With advancing age, cellular functions such as the DNA repair, epigenetic modifications, telomere activity, protein folding, mitochondrial respiration, stem cell regeneration, and cellular communication are deviated from their physiological condition, leading to systemic proinflammation. The inflammatory regulatory cascades are the key signaling that drives the cellular changes involved in the adverse arterial wall remodeling thus facilitating the exponential increase in mortality and morbidity related to hypertension and atherosclerosis. In this research topic, titled “arterial aging and age-related arterial diseases,” the views of the physiological and biochemical mechanisms that affect vascular proinflammation and inflammatory diseases with aging have been updated. Additional original research articles on molecular and cellular mechanisms, diagnosis, and treatment of arterial adverse remodeling associated with aging have also been included.

### Metabolism disorders and aging

With advancing age, the glucose metabolism in arterial wall disorders due to insulin resistance, declined growth hormone, and cellular senescence (Strazhesko et al.). Aging increases the deposits of the advanced glycation end-products (AGEs), the long-lived molecules of advanced glycation of extracellular matrix in the basement membrane and interstitial space of cells (Senatus and Schmidt). The basement is thus glycated and as result becomes stiffen and fractured (Senatus and Schmidt). The disrupted and modified basement membrane promotes endothelial proinflammation and dysfunction as well as the synthetic phenotypic shift of vascular smooth muscle cells (VSMCs) (Senatus and Schmidt). The glycated interstitial AGEs interact with their receptor RAGE of vascular cells initiating a cascade of events leading to proinflammation and stiffening of the vasculature (Senatus and Schmidt). Importantly, aging increases the prevalence of the metabolic syndrome and modifies blood coagulation factors increasing vascular inflammation and the risk of thrombosis and atherosclerosis (Lagrange et al.). In addition, apolipoprotein E (APOE) gene exerts important roles in the regulation of lipoprotein metabolism, and its mutation is closely associated with an increased risk of coronary artery disease in individuals with metabolic disorder (Luo et al.).

### Inflammation, calcification, and arterial stiffening

Arterial stiffness is a determinant of arterial biological aging rather than arterial chronological aging due in large to genetic and epigenetic factors leading to inflammation and calcification. Aging increases Angiotensin II (Ang II) pro-inflammatory signaling in the arterial wall. Ang II modifies the levels of several micro RNA (miR) including miR 21, 22, and 29 (Duggirala et al.; Nanoudis et al.). miR-21 increases arterial fibroblasts survival and extracellular matrix (ECM) deposition (Cheng et al., 2016). miRs-22 promotes VSMC phenotypic shifts from contractile to synthetic phenotype. miR-29 affects the synthesis of elastin and different types of collagen. Thus, Ang II signaling alters the arterial proinflammatory niche modified by miRs, leading to arterial ECM remodeling (Nanoudis et al.).

Arterial calcification is a sequelae of calcium metabolism disorder and inflammation of vascular wall (Nanoudis et al.). Trans-differentiation of VSMCs to a chondrocyte or osteoblast-like phenotype is the main cellular way by which arterial calcification can lead to arterial stiffening (Nanoudis et al.). The medial calcification precedes the atherosclerotic plaque development (Nanoudis et al.). Notably, miRs affect arterial calcification through miR-29-elicited matrix metalloproteinase type II (MMP-2) activation (Nanoudis et al.) and calcium deposition and osteoblast differentiation (Nanoudis et al.).

Arterial stiffening or the extracellular-associated stiffening markedly affects the function of endothelial cells with aging (Kohn et al.). Changes in endothelial cells or the surrounding ECM directly impact endothelial health, such as nitric oxide (NO) production and barrier integrity against leukocyte transmigration (Kohn et al.). VSMCs are durotactic and preferentially migrates toward increased stiffness substrates and ECM chemical cues and proliferates on stiffer matrixes (collagen) (Kohn et al.).

### Arterial stiffening and hypertension

The age-associated increase in arterial stiffness is well correlated with the incidence of hypertension. In this issue, Kohn et al. comprehensively reviewed the methods of measurement of arterial stiffness at both the macro-and microscale, including the pulse wave velocity macroscale measurement in clinic and microscale atomic force microscopic measurements in the research laboratory (Kohn et al.). As a unique advantage for large epidemiological studies, this issue analyzes the mechanical defects due to arterial aging based on the measured aortic pressure and an assumed triangular flow, paving the way to consider the clinical application of the simplified method for estimating the arterial stiffness (Chang et al.). Arterial intima stiffening measurements have highlighted that hypertension is preceded by arterial stiffening, and elevated arterial stiffening preceded increases in blood pressure in subjects with rheumatoid arthritis (Woodman et al.). This effect is abolished by methotrexate treatment (Woodman et al.).

### Aging atherosclerosis

Atherosclerosis is driven by aging (Head et al.). The mummies studies suggest that atherosclerosis is not a lifestyle dependent disease, rather than, an aging driven disease (Head et al.). Atherosclerosis is a chronic inflammatory, progressive disease with manifestation starting at a young age in children under the age of 10 years and even <1 year old (Head et al.). Aging promotes the dynamic changes of monocytes/macrophages and disorders of lipid. Elderly macrophages assist the living of atheroma and promote the formation of plaques (Head et al.).

### Aging stroke

The incidence of stroke is an early pro-thrombotic phenotype of atherothrombotic events with aging. Such an aspect is reinforced by the pro-coagulant properties of de-differentiated and pro-inflammatory VSMCs due to their increased fibrinogenesis and impaired fibrinolysis and increased saturated fatty acids (Lagrange et al.). Atherothrombotic events are also associated with the metabolic syndrome (Lagrange et al.).

### Aging arterial dissection/aneurysms

The dissection and aneurysm are the deadly complications of hypertension and atherosclerosis in the elderly population. Non-coding RNAs, including micro-RNAs and long non-coding RNAs, are recently emerging as important modifiers of gene transcription and cell function (Duggirala et al.). Non-coding RNAs such as miR-21, 29, and 34 are alerted in the arterial wall and involved in the development of arterial aneurysm (Duggirala et al.). Milk fat globule EGF-VIII fragment medin-amyloid, a characterized age-associated arterial amyloid, plays a role in the process of hypertension, atherosclerosis, and aneurysm and dissection (Wang et al.).

### Aging peripheral arterial disease

Aging is the major risk factor for the incidence of peripheral arterial disease accompanied by an increase in pulse pressure. Mao et al. performed a prospective cohort observation and investigated the association of pulse pressure with peripheral arterial disease incidence in an elderly general population and they found that the hazard ratio for people with pulse pressure more than 60 mmHg was 2.20 compared with those whose pulse pressure was <40 mmHg (Mao et al.).

### Aging hypertension

Chronic hypertension is one of the most common aging-related disorders. The impairment of endothelial cells function along with the alteration in VSMCs calcium homeostasis plays a fundament role in the development of hypertension. In their review, Eid et al. analyze and discuss the role of different type of Inositol 1,4,5-Trisphosphate Receptors (IP_3_R) in the onset and progression of hypertension via their effect on VSMCs physiological functions, such as vascular tone regulation and VSMCs phenotypic switch to proliferative phenotype in age-associated vascular remodeling (Eid et al.).

### Anti-aging molecules

Hydrogen sulfide (H_2_S), like a nitric oxygen (NO), may be an anti-inflammatory vascular gaseous mediator. Endogenous H_2_S is produced during the cysteine metabolism, which is regulated by the pyridoxal phosphate-dependent enzymes cystathionine β-synthase (CBS) and cystathioine y-lyase (CSE). CSE KO in mice reduces the production of H_2_S and predispose the animals to the development of early atherosclerotic lesions, however, supplement of H_2_S effectively alleviates the initiation and progression of atherosclerosis (Lin et al.). A further study show that H_2_S supplementation in to ApoE^−/−^ mice promotes the expression of Ang II converting enzyme 2 (ACE2), resulting in a decrease of Ang II in endothelial cells, and eventually diminishing the prevalence and severity of atherosclerosis (Lin et al.).

Apelin is synthesized as 77-amino acid preproapelin and rapidly cleaved into active isoforms, including apelin-13, which is predominantly enriched in the cardiovascular system (Zhou et al.). Apelin-13 is a negative regulator of aging-mediated and Ang II-mediated adverse cardiovascular remodeling and dysfunction via an activation of the apelin receptor (Zhou et al.). Apelin 13 receptor signaling mediates the endothelial-dependent vasodilation (Zhou et al.).

### Anti-aging lifestyles

The regular aerobic exercise effectively alleviates the age-related symptoms and signs. In this issue, Chen et al. report that the habitual exercise improves the aortic endothelial mitochondria function via the activation of an adenosine monophosphate-activated protein kinase α2 (AMPK α2) (Chen et al.). In addition, exercise also improves the trunk flexibility, known as an index of the body stiffness. The flexibility is a predictor of arterial stiffness (Gando et al.). The improvement of the body flexibility reduces the progression of arterial stiffening with aging (Gando et al.).

## Author contributions

All authors listed have made a substantial, direct and intellectual contribution to the work, and approved it for publication.

### Conflict of interest statement

The authors declare that the research was conducted in the absence of any commercial or financial relationships that could be construed as a potential conflict of interest.

## References

[B1] Cheng M. Wu G. Song Y. Wang L. Tu L. Zhang L.. (2016). Celastrol-induced suppression of the MiR-21/ERK signalling pathway attenuates cardiac fibrosis and dysfunction. Cell. Physiol. Biochem. 38, 1928–1938. 10.1159/000445554 27160852

